# Process evaluation of a multi-disciplinary complex intervention to improve care for older patients with chronic conditions in rural areas (the HandinHand Study): study protocol

**DOI:** 10.1186/s12912-022-00858-6

**Published:** 2022-06-14

**Authors:** Swantje Seismann-Petersen, Sascha Köpke, Simone Inkrot

**Affiliations:** 1 Institute of Nursing Science, University of Cologne, Faculty of Medicine and University Hospital Cologne, Gleueler Str. 176-178, 50935 Köln, Germany; 2 Institute for Social Medicine and Epidemiology, Nursing Research Section, University of Lübeck, Ratzeburger Allee 160, 23562 Lübeck, Germany

**Keywords:** Process evaluation, Complex intervention, Logic model, Mixed methods, Study protocol, Primary care, Nursing, Advanced nursing practice, Role development

## Abstract

**Background:**

To improve health care in rural areas, especially for increasing numbers of people with chronic diseases, academically qualified nurses could take over expanded roles to meet the challenges of an ageing society and a decreasing number of General Practitioners (GPs). In the project “HandinHand” (HiH), qualified nurses (Expert nurses, ENs) will carry out home visits to older people with chronic diseases over a period of six months. ENs will prepare a care plan in cooperation with GPs to stabilise the care situation and avoid unplanned hospital admissions and GP visits. The process evaluation aims to provide an in-depth analysis of the implementation process and gather important information on barriers and facilitators to the implementation of ENs as a complementary health care structure in primary care, taking into account several context factors.

**Methods:**

Based on the Medical Research Council (MRC) Framework for complex interventions, a logic model was developed and applied as the basis for data collection. Qualitative and quantitative data will be collected during the study. A mixed methods approach should allow to gain important insights from participants (e.g. ENs, GPs, patients) involved in the study as well as relevant stakeholders. Semi-structured interviews and surveys will be conducted. Data analysis will be based on the logical model, combining qualitative and quantitative data. Qualitative data will be analysed inductively-deductively using qualitative thematic framework analysis.

**Discussion:**

The process evaluation will provide guidance and conclusions on further development and transferability. Of particular interest is the expanded role of ENs in primary care, which has barely been implemented in Germany and can be seen as a precursor to the development of an Advanced Practice Nursing (APN) role in primary care.

## Background

Demographic change, increasing health care needs and consumer expectations highlight the need for transforming and restructuring healthcare services worldwide [26]. With a growing population of people over 60, healthcare systems are faced with challenges to meet growing demands within a context of a shortage of healthcare professionals. Most older people are able to care for themselves, but in later life many also suffer from one or multiple chronic illnesses and disabilities and require support from healthcare professionals. Health care needs of persons with chronic health conditions are multi-fold and complex and cannot be adequately met by one health profession alone. Rather, care models involving multidisciplinary teams and coordinating services to include a mix of skills and competencies tailored to the complex needs of persons with chronic conditions have shown to improve outcome [15, 21].

Particularly for people in rural areas, many countries have responded to growing demands for multidisciplinary care models acknowledging evidence of the importance of person-centred care approaches enabling support and empowerment for self-management at home for people with chronic conditions [8, 19]. In many countries, models of community-based, ambulatory care have been proposed instead of models of acute medical care provided mostly by hospitals aiming to increase health care access and reduce potentially avoidable hospitalisations [20, 21].

In primary care, the effectiveness of traditional, physician-driven models have been questioned and alternative models were successfully implemented [15]. Collaborative, integrated care models have been shown to have a positive impact on a number of patient outcomes in a variety of primary care settings [21]. New opportunities have emerged for nurses, especially advanced practice nurses (APN), to meet patients’ demands and unmet needs [1–4, 11, 19]. Internationally, APN claim essential roles as practitioners within health care systems and are in a prime position to drive innovative care models to improve the coordination of multidisciplinary health care for older people [19]. Advanced nursing practice has been defined as “patient-focused application of an expanded range of competencies to improve health outcomes for patients and populations in a specialized clinical area of the larger discipline of nursing.” (Tracy & O´Grady, [30]). Current evidence suggests that when APN are competent and empowered within the health system to practice as independent professionals, their work is associated with improvements in several measures of health outcomes and behaviour and leads to comparable or better results compared to care provided by medical doctors alone [17, 18].

In Germany, the establishment of educational and practice opportunities for nurses to become APN started later and developed slower than in many other Western countries, and the German health system still faces important legislative, professional and structural barriers that prevent APNs (or indeed any nurse) to practice as independent professionals. A re-organisation of health care provision and greater autonomy for nursing practice has been recommended by various legislative and professional bodies in Germany for many years but so far, the legal framework for such independent provision of healthcare by nurses is lacking [6, 27, 28]. In Germany, nurses are not entitled to bill for healthcare provided, refer patients to other health professionals, or prescribe drug or nursing aids. Nurses are authorised to practice medical care e.g. wound care management only upon delegation and referral from medical professionals. Also, academic educational opportunities for nurses beyond basic training in the form of tertiary education have developed much later than in most developed countries. Hence, from a global perspective, the development of APN roles and opportunities in Germany is clearly delayed and there is a dire need for concerted efforts to increase evidence-based advanced and expanded practice opportunities.

The “HandinHand” (HiH) study, funded by the German Innovation Fund of the Joint Federal Committee [9], addresses the development and evaluation of expanded nursing roles within an innovative, multidisciplinary care model in primary care. The study will investigate the effects of a complex intervention that aims to improve outcomes for community-dwelling older people with chronic conditions. The core of this intervention will be the implementation of an Expert Nursing Centre (ENC), comprising a team of qualified nurses with expanded practice competencies and additional tertiary nursing education at Bachelor-level working in collaboration with general practitioners (GPs) and other health care professionals (HCP). Successful development, implementation and utilisation of any new role is complex, particularly in multidisciplinary teams [29]. Therefore, a comprehensive process evaluation will be undertaken alongside the main study to explore barriers and facilitators regarding implementation, mechanisms of impact and the context of the study. This paper describes the process evaluation, including design and methods.

## Methods

### Aims

The overall aims of the process evaluation are to explain discrepancies between expected and observed results, identify how potential mechanisms of impact and the context influence the results, and provide insights to support further development and future implementation of the intervention.

### Setting

The HiH intervention will be led by a core study team that consists of the principal investigator (PI), a project manager, 10 expert nurses (ENs), a nursing team leader and a co-leader (EN team leader), and an administration assistant. The wider study consortium will consist of eight institutions, whose responsibilities are shown in Table 1. An external advisory board, led by the University of Cologne, with representatives from various stakeholders (patients, nurses, physicians, scientific experts, educational institutions, health insurances) will be responsible for overseeing and regularly advising on the conduct of the study. The University of Lübeck and the University of Cologne will be responsible for the process evaluation.

**Table 1:** Study consortium

Institution	Responsibility
1. Marienhausholding GmbH	Project management
2. RWI—Leibniz Institute for Economic Research	Data collection and outcome evaluation
3. University of Lübeck	Data collection and process evaluation
4. University of Cologne	
5. Rechenzentrum Volmarstein GmbH, RZV	Provision of an electronic patient documentation system (ePa)
6. Philosophisch-Theologische Hochschule Vallendar (Catholic University)	Education of ENs at Bachelor-level
7. AOK Rhineland-Palatinate/Saarland, Health Insurance	Provision of comparative data
8. Medical Association Ahrweiler	Cooperation with physicians in the region

In the HiH study, patients over 60 with multiple chronic conditions and complex care needs will be identified and recruited by their GPs and, following informed consent, referred to the team of ENs, who will be based at an independent ENC specifically set up for the purpose of the study. The ENs will be provided with a description of the individual patients’ health needs and GPs will delegate specific interventions to be carried out by the ENs. As part of their first home visit, the ENs will perform a comprehensive health assessment and will develop a care plan together with the patients and their relatives, adding to the list of care needs identified by the GP as required. ENs will visit the patients in their home at least monthly, for six months, with the aim of providing timely access to health care, improving care coordination with other providers, relatives and community networks, and supporting self-management and stabilisation of the patients’ health situation. The main anticipated outcomes of the HiH study are reduced number of GP home visits, and reduced number of avoidable hospitalisations over a period of three years. Patient-reported outcomes include quality of life and satisfaction with care received.

The process evaluation will be undertaken in consultation with the external advisory board. The board will be informed about the progress of the process evaluation on a three-monthly basis via face-to-face/digital/phone conferences alternating as required and will provide feedback regarding its progress and the interpretation of interim results. The board will also be involved in designing data collection instruments as per individual board members’ expertise. The process evaluation team will also work closely with the HiH core study team, as well as the wider project consortium such as the teams responsible for data management and outcome evaluation. This will reduce the risk of duplicate data collection.

### Design

The theoretical basis of the process evaluation is based on the Medical Research Council (MRC) Framework for the process evaluation of complex interventions [23]. Given the intervention’s complexity, several dimensions of influence need to be considered and evaluated in order to provide recommendations for further development or adaptation of the intervention and/or its implementation into practice. The outcomes of a complex intervention cannot be determined by single, simply reproducible actions alone and are influenced by the interplay of several factors. These include the theoretical background of the intervention, the intervention itself, the implementation of the intervention, mechanisms of impact (which in turn are all influenced by providers and recipients of the interventions), and the context in which the intervention will be delivered. The purpose of a process evaluation is to highlight individual components of each of these factors during a study investigating a complex intervention. The results can help determine why and how this complex intervention has led to certain outcomes and hence provide transferrable conclusions and guidance on further development and implementation.

Therefore, this process evaluation aims to collect data in order to:

- describe the content of the intervention, and dose, reach, fidelity and adaptations of the implementation.
- identify mediator variables for the development of the effect of the intervention.
- identify relevant contextual factors for the implementation of the intervention.
- describe process outcome parameters.

**Fig. 1:**
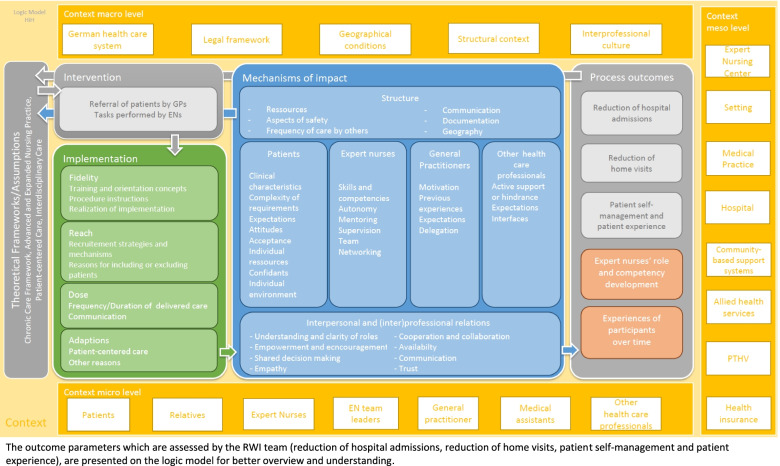
Logic model

Prior to planning the specific data collection trajectory and instruments, a logic model to clarify and specify relevant theoretical assumptions on which the intervention is based, was developed. The logic model states assumptions underpinning the assumed effect, the intervention itself, the implementation of the intervention, potential mechanisms of impact, outcomes, and the context in which the intervention is delivered [16, 23] (Fig. 1). Relevant literature was considered to develop the logic model in collaboration with the core study team, the wider consortium, and the external advisory board, who all provided recommendations for refinement of the model.

### Sample and sample size

As the purpose of the process evaluation is to collect information on different research questions from diverse perspectives, data collection will be conducted with different participants and stakeholders involved in the study. Therefore, the target groups for qualitative and quantitative data collection will be all ENs employed at the time of data collection (n = 10 at the beginning of the study), EN team leaders (n = 2), the principal investigator (n = 1) and GPs involved in the study (n = to be specified). In order to represent the perspective of those indirectly or directly affected by the project, interviews will be conducted with randomly selected patients (n = 18 over the course of the study) and relatives (n = 18 over the course of the study) as well as health care professionals (n = 5 at each point of data collection) from other health care institutions in the region involved in the project. Sample sizes have been determined pragmatically taking into account the potential burden for participants. In addition, the perspective of the health insurance as a relevant stakeholder and that of the PTHV as provider and implementer of the Bachelor's programme are of interest, thus, two representatives of each institution will be interviewed at different times during the course of the project.

### Data collection

Qualitative data will be collected face-to-face, by telephone or video-telephony, recorded verbatim and transcribed, with data anonymised during the transcription process. All data will be stored anonymously, only date and time of measurement will be documented, and will only be analysed at group level. Contact with patients and relatives will be coordinated via the EN team leader, so that personal data will only be used for contact after a declaration of consent has been obtained. Quantitative data (questionnaires of ENs, EN team leaders, PI, GPs) will be collected online using Lime Survey (LimeSurvey Community Edition, Version 3.27.2), quantitative patient-related data will be collected in the electronic patient documentation system (ePa) and made available by RWI.

**Fig. 2:**
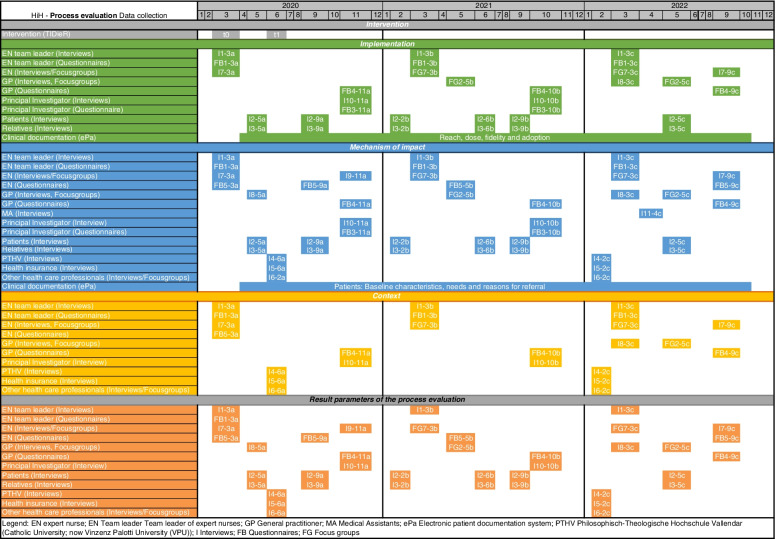
Gantt-Chart of data collection

Following the identification of key concepts and important factors, the data collection plan was developed (Fig. 2).

**Fig. 3:**
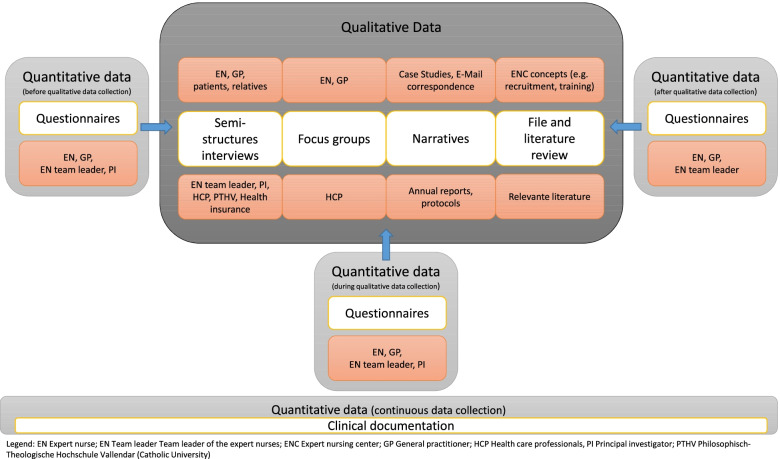
Embedded mixed-methods approach for the HiH Study [5]

To answer the different research questions and to get a better understanding of the effect of the intervention, a mixed-methods approach consisting of quantitative (questionnaires, clinical documentation) and qualitative (semi-structured interviews, focus groups, narratives, files/literature reviews) methods is used. This can be classified as an embedded approach in a mixed methods intervention design following Creswell & Plano Clark [5] (Fig. 3). Thus, quantitative Data will be collected and analysed before, during, and after the qualitative data collection. Narratives provided by the core study team in the form of reports, protocols, case studies and E-Mail correspondence will be collected and additionally assigned to the constructs of the logic model. Data collections are planned at the beginning, during, and at the end of the study aiming to capture potential developments and changes over time.

Based on the logic model, the process evaluation is planned to provide an adequate number of data collections during the implementation of the intervention incorporating the perspective of all relevant groups of persons (GP, EN, patients, relatives, EN team leader, principal investigator) and stakeholders (representatives of health insurance and professional bodies as well as other health care professionals). This will provide sufficient information to allow for a comprehensive description of different mechanisms of action and achieve data saturation.

In line with the MRC framework [23], the details of data collection and content in each of the process domains and indicators are described below. All adjustments and changes in data collection (time points/methods) will be recorded with reasons in a tabular overview.

1. Intervention, theoretical frameworks and assumptions
  - Development process
    This will include a description of the intervention development processes carried out by the HiH core study team, including theoretical foundations and consensus building in cooperation with the wider project consortium and members of the advisory board. The process included a first consensus of the intervention with a refinement after four months of intervention implementation that led to the final description of the intervention. This step was necessary to allow for further specification and adaptation of the individual intervention components to the clinical and sociodemographic characteristics of included patients and a better understanding of their needs.
  - Description of the intervention
    The components of the intervention were described in detail using the “Template for Intervention Description and Replication” (TIDieR) checklist and guide [12]. Details of data collections related to the intervention are described in Table 2.

**Table 2:** Data collection in the process domain “intervention”

Process domain indicator	Topic	Data sources	N	Data collection methods	Operationalisation/ Parameters	Data type	
						Quant	Qual
a. Development process	Intervention development	TIDieR Checklist first version, meeting protocols (Advisory Board; research team/HiH Core team)	n.a	File review	Challenges in specifying the intervention Adjustments to the intervention made between t0 and t1 Theoretical foundations		descript-tive
b. Description of the intervention	HiH Intervention	TIDieR Checklist final version	n.a	File review	Components of the intervention		descript-tive

n.a. not applicable
2. Implementation
  In this section, aspects of reach, dose, fidelity and adaptation will be explored. Details of data collections pertaining to the process domain of implementation are described in Table 3.
  - Fidelity
    Fidelity will include the extent to which the intervention was implemented as planned. The training and orientation concepts will be described, including the necessary skills and competencies as well as how these are acquired.
  - Reach
    This refers to the extent to which the intended population will come into contact with the intervention. It will include strategies both to recruit participating GPs as well as recruitment strategies to include patients into the study and reasons for excluding patients at GP level. Barriers and facilitators of recruitment and strategies to address challenges with recruitment will be explored.
  - Dose
    This essentially will refer to the quantity of the intervention delivered. Data on the frequency and duration of care will be collected. Information will also be collected on communication channels and types of communication that directly affect care (particularly communication between the expert nurses and GPs).
  - Adaptations
    This will describe alterations and modifications to the intervention on an individual basis to achieve better contextual fit. Reasons for person-centred adaptations will be described.

**Table 3:** Data collection in the process domain “implementation”

Process domain indicator	Topic	Data sources	N (planned number of persons per data collection)	Data collection methods (number of planned data collections where applicable)	Operationalisation/ Parameters	Data type	
						Quant	Qual
**Fidelity**	Initial orientation/ training and ongoing professional development	HiH Training concept	n.a	File review	Structure and content of initial orientation/ training and ongoing professional development		descriptive
		EN team leader	1 2	Questionnaire (3), interviews (3)	Frequency and individual content of initial orientation/ training and ongoing professional development	✓	✓
	Competencies	HiH project team documentation	n.a	File review	Required competencies and how these are acquired		descriptive
	Interventions requested/ planned and interventions implemented	ePA	All patients	Clinical documentation	Content and number of referral requests Number and type of additional needs identified Content and number of interventions implemented	✓	
	Establishment of the ENC service	ENs	10	Interviews (2)	Barriers and facilitators for ENC service establishment		✓
		EN team leader	2	Interviews (3)	Barriers and facilitators for ENC service establishment		✓
		Principal investigator	1	Interviews (2)	Barriers and facilitators for ENC service establishment		✓
**Reach**	Recruitment strategies and mechanisms	Principal investigator	1	Questionnaires (2), interviews (2)	Methods of recruitment incl. barriers and facilitators	✓	✓
		EN team leader	1 2	Questionnaires (3), interviews (3)	Methods of recruitment incl. barriers and facilitators	✓	✓
		GPs	All GPs	Questionnaires (3), focus groups (1)	Methods of recruitment incl. barriers and facilitators	✓	✓
		HiH Recruitment concept	n.a	File review	Recruitment strategies		descriptive
		Patients	2–4	Interviews (6)	How do patients become aware of the study and possibility of participation		✓
		Relatives	2–4	Interviews (6)	How do relatives become aware of the study and possibility of participation		✓
	Patient selection	GPs	All GPs	Questionnaires (3)	Number of eligible patients, how many were excluded and why	✓	
**Dose**	Frequency of care	ePA	All patients	Clinical documentation	Number of visits	✓	
	Duration of care	ePA	All patients	Clinical documentation	Duration of - direct patient care - indirect patient care - travel time	✓	
**Adaptations**	Person-centred adjustments to the specific interventions	ePA	All patients	Clinical documentation	Content and number of adjustments of interventions with reasons	✓	
		Narratives	n.a	Case studies	Context in which the intervention was adapted		

*EN* Expert nurse, *ENC* Expert nursing centre, *EN Team leader* Team leader of the expert nurses, *ePA* Electronic patient documentation system, *GP* General practitioner, *HiH* HandinHand study, *Quant.* Quantitave, *Qual.* Qualitative
3. Mechanisms of impact
  In this section, the overarching mediator variables identified in the development of the logic model are described according to the following thematic foci: structure, (core)participants, interpersonal and (inter)professional relationships. Details of data collections pertaining to the process domain of mechanisms of action are described in Table 4.
  - Structure
    As it can be assumed that structural conditions influence the intervention, the following aspects are identified as relevant and thus will be investigated:
    Resources available to the EN and their team leader (e.g. material equipment as well as Standard Operating Procedures)
    Aspects of safety regarding practice in rural areas; emergency management, availability of supporting system
    Frequency of care provided to participating patients by other caregivers (formal/informal)
    Communication and documentation structures (e.g. formal communication structures; documentation of patient data in an electronic documentation system)
    Geographical conditions/special features of the catchment area (e.g. size of the catchment area, distances between ENC and patient homes, availability of data infrastructures).
  - Core Participants
    Core participants are directly involved in the intervention. These include the participating patients and GPs, the ENs and other persons involved in the care of the patients, e.g. relatives, nurses in nursing homes or other health care professionals. In Table 4, it can be seen that, depending on the topic, data collection will be conducted with both core participants and other relevant stakeholders and people involved in the intervention.
    1. Patients
      In addition to describing baseline characteristics, self-reported needs, expectations, and preferences regarding participants’ health care in general and the care provided by ENs, as well as individual resources and coping strategies, and patient acceptance of ENs will be assessed.
    2. Expert Nurses
      As the expanded role of nurses in primary care in Germany is new and has therefore not been well studied, role and competency development, as well as the associated barriers and facilitators, will be a focus of the research. In this context, it is of interest how the ENs as well as the GPs will describe the degree of perceived autonomy. As aspects such as mentoring, supervision, team and networking are described as relevant factors of role development [14, 29], these will also be investigated.
    3. General Practitioners
      Physicians' motivation to participate in the project, whether and what experience they will have regarding collaboration with nurses with expanded roles, their expectations, and their attitudes toward delegating tasks to ENs will be explored.
    4. Other health care professionals
      The perspective of other health care professionals on a new role in the health care system as well as their experience with taking over this new role will be explored. Therefore it will be investigated what expectations exist, what interfaces will be described and how they will handle them. Furthermore, trying to identify factors that promote or hinder role development will be another aim.
  - Interpersonal and (inter)professional relationships
    The interpersonal and (inter)professional relationships between all participants can be counted as important mechanisms of impact [7, 24, 26, 29]. Therefore, the relationships between ENs and patients, ENs and relatives as well as the relationships between ENs and GPs, within the EN team and with other health care professionals will be examined. In this context, the focus will be on the following criteria:
    Role understanding and role clarity; e.g. the understanding of the role and responsibilities, and differences to other roles in the health care systems
    Encouragement and empowerment; e.g. to what extend the ENs perceive support and empowerment, but also to what extend patients and relatives describe perceived encouragement
    Shared-Decision making; e.g. to what extend ENs feel confident to involve patients in care planning und how patients/relatives perceive involvement
    Empathy; e.g. if and how patients and relatives perceive empathy
    Cooperation and collaboration; e.g. experienced cooperation between health care professionals and what factors they describe as facilitating and hindering
    Accessibility; e.g. how ENs and GPs assess mutual accessibility
    Communication; e.g. communication experienced between ENs and GPs, ENs and EN team leader, within the EN team as well as between ENs and patients/relatives
    Trust; e.g. whether patients/relatives describe the relationship with the ENs as trusting

**Table 4:** Data collection in the process domain “mechanisms of impact”

Process domain indicator	Topic	Data sources	N (planned number of persons per data collection)	Data collection methods (number of planned data collections where applicable)	Parameters/ Operationalisation	Data type	
						Quant.	Qual.
a. Structure	Resources	EN team leader	1 2	Questionnaires (3), interviews (3)	Teamwork & networking Description of collaboration with University	✓	✓
	Safety	EN team leader	1 2	Questionnaires (3), interviews (3)	Assessment of aspects of safety regarding practice in isolation & home visits Availability of supervision & mentoring Emergency management	✓	✓
	Catchment area & geography	EN team leader	1 2	Questionnaires (3), Interviews (3)	Size of the areas in qm2 Particular geographic features (e.g. poor road conditions, lack of mobile coverage)	✓	✓
		ePA	All patients	Clinical documentation	Distance between nurses’ office and patient homes in km	✓	
	Documentation and communication	Principal investigator	1	Questionnaires (2), interviews (2)	Features of communication within the team and externally incl. barriers and facilitators	✓	✓
		EN team leader	1 2	Questionnaires (3), interviews (3)	Structure and features of communication and clinical documentation incl. barriers and facilitators	✓	✓
		ENs	10	Interviews (3), questionnaires (3), focus groups (2)	Structure and features of communication and clinical documentation incl. barriers and facilitators	✓	✓
		GPs	All GPs	Questionnaires (3)	Use of the referral document and perceived usefulness Structured/systematic communication with ENs, barriers and facilitators	✓	
b. Core participants							
**Patients**	Baseline characteristics	ePA	All patients	Clinical documentation	Age, sex, living situation, diagnoses etc	✓	
	Needs	Patients	2–4	Interviews (6)	Self-reported needs		✓
		Relatives	2–4	Interviews (6)	Self-reported patient and own needs		✓
		ePA	All patients	Clinical documentation	Reasons for referral GP-reported patient needs EN-reported patient needs	✓	
		ENs	10	Questionnaires (3)	EN-reported patient needs	✓	
	Expectations and preferences	Patients	2–4	Interviews (6)	Expectations and preferences regarding own health care in general and care by the ENs		✓
		Relatives	2–4	Interviews (6)	Expectations and preferences regarding patient’s health care in general and care by the ENs		✓
	Acceptance	Patients	2–4	Interviews (6)	Indications of the extent to which the role of ENs is recognised as a complementary role in the care system		✓
		Relatives	2–4	Interviews (6)	Indications of the extent to which the role of ENs is recognised as a complementary role in the care system		✓
	Resources	ePA	All patients	Clinical documentation	Other health care and allied services involved	✓	
		Patients	2–4	Interviews (6)	Individual resources & coping strategies		✓
**ENs**	Skills and competencies	ENs	10	Questionnaires (3), interviews (3), focus groups (2)	Own perceptions of skills and competencies	✓	✓
		EN team leader	1 2	Questionnaires (3), interviews (3)	Perceptions of ENs competencies	✓	✓
		GPs	All GPs	Questionnaires (3), interviews (2)	Perceptions of ENs competencies, task sharing	✓	✓
		PTHV	2	Interviews (2)	Perceptions of ENs competencies		✓
		Health insurance representatives	2	Interviews (2)	Perceptions of ENs competencies		✓
	Autonomy	ENs	10	Questionnaires (4), interviews (3), focus groups (2)	Perception of autonomy	✓	✓
		GPs	All GPs	Questionnaires (3), interviews (2), focus groups (1)	Perception of ENs autonomy	✓	✓
	Mentoring & Supervision	ENs	10	Questionnaires (3), interviews (3), focus groups (2)	Needs and perception of mentoring and supervision	✓	✓
		EN team leader	1 2	Questionnaires (3), interviews (3)	Strategies for mentoring and supervision Perception of ENs needs	✓	✓
	Team	ENs	10	Questionnaires (3), interviews (3), focus groups (2)	Team as a resource Teamwork	✓	✓
		EN team leader	1 2	Questionnaires (3), interviews (3)	Team building strategies	✓	✓
	Networking	EN team leader	1 2	Questionnaires (3), interviews (3)	Type and structure of networking Barriers and facilitators of networking	✓	✓
**GPs**	Motivation	GPs	All GPs	Questionnaires (3), interviews (2)	Reasons for participation in the study	✓	✓
	Previous experiences	GPs	All GPs	Questionnaires (3), interviews (2)	Experiences working with nurses with expanded roles	✓	✓
	Expectations & experiences	GPs	All GPs	Questionnaires (3), interviews (2), focus groups (2)	Expectations and experiences regarding interprofessional cooperation and competencies of ENs	✓	✓
	Delegation	GPs	All GPs	Interviews (2)	Delegated tasks and whether they have changed	✓	✓
**Other health care professionals**	Expectations	Health care professionals from other health care services involved in HiH patient care	5	Interviews (2)	Expectations regarding cooperation		✓
	Active support and hindrance	Health care professionals from other health care services involved in HiH patient care	5	Interviews (2)	To what extent the project/participation in the project is recommended to other patients/GPs/relatives		✓
	Interfaces	Health care professionals from other health care services involved in HiH patient care	5	Interviews (2)	Expected interfaces and how to handle them		✓
c. Interpersonal and (inter)professional relationships	Role understanding, role clarity	ENs	10	Questionnaires (4), interviews (3), focus groups (2)	Understanding of role performance, responsibilities and tasks Differences to other health care professionals	✓	✓
		EN team leader	2	Interviews (3)	Understanding of role performance, responsibilities, tasks, needed competencies Differences to other health care professionals	✓	✓
		GPs	All GPs	Questionnaires (3), interviews (2), focus groups (1)	Understanding of role performance, responsibilities and tasks Differences to other health care professionals	✓	✓
		Patients	2–4	Interviews (6)	Understanding of EN role, responsibilities and tasks Differences to other health care professionals		✓
		Relatives	2–4	Interviews (6)	Understanding of EN role, responsibilities and tasks Differences to other health care professionals		✓
		Other health care professionals from other health care services involved in HiH patient care	5	Interviews (2)	Understanding of EN role, responsibilities and tasks Differences of EN to other health care professionals		✓
		PTHV	2	Interviews (2)	Understanding of EN role, responsibilities and tasks Differences to other nurses with expanded roles/to ANP/to other health care professionals		✓
	Empowerment & encouragement of ENs	EN team leader	2 1	Interviews (3), questionnaires (3)	Measures for the professional development of the EN	✓	✓
		PTHV	2	Interviews (2)	Needed EN competencies Measures to support role identification		✓
		GPs	All GPs	Questionnaires (3), interviews (1)	Support EN in performing/taking over delegated activities		✓
		ENs	10	Interviews (2), questionnaires (3)	Perceived encouragement/empowerment by GPs, EN team leader, other EN, PTHV	✓	✓
	Empowerment & encouragement of patients/relatives	Patients	2–4	Interviews (6)	Description whether and how patients feel supported by the ENs in dealing with disease-related challenges in daily life		✓
		Relatives	2–4	Interviews (6)	Description whether and how relatives feel supported by the ENs in caring for their patient		✓
		ENs	10	Focus group (1), questionnaires (3)	Perceived contribution of ENs to patient self- and symptom management	✓	✓
	Shared decision making	Patient	2–4	Interviews (6)	Description whether and how patients/relatives feel involved in the planning and delivery of individual care		✓
		Relatives	2–4	Interviews (6)	Description if and how patients/relatives feel involved in the planning and delivery of individual care		✓
		ENs	10	Questionnaires (3), interviews (2)	To what extent the ENs feel confident to involve patients/relatives in identify care needs and develop individual care plans	✓	✓
	Empathy	Patients	All Patients	Outcome evaluation questionnaires	Perceived empathy	✓	
	Cooperation and collaboration	ENs	10	Questionnaires (4), interviews (3), focus groups (2)	Expectations, experiences Hindering and facilitating factors	✓	✓
		GPs	All GPs	Questionnaires (3), interviews (2), focus groups (2)	Expectations and experiences regarding cooperation with ENs and EN team leader	✓	✓
		Other health care professionals from other health care services involved in HiH patient care	5	Interviews (2)	Expectations and experiences		✓
		EN team leader	2 1	Interviews (3), questionnaires (3)	Measures to support internal and external cooperation /collaboration Barriers and facilitators	✓	✓
	Accessibility	ENs	10	Questionnaires (3)	Assessment of accessibility of GPs	✓	✓
		GPs	All GPs	Questionnaires (3)	Assessment of accessibility for ENs and accessibility of ENs	✓	✓
		Patients	2–4	Interviews (6)	Accessibility of the ENs		✓
		Relatives	2–4	Interviews (6)	Accessibility of the ENs		✓
	Communication	ENs	10	Interviews (3), focus groups (2), questionnaires (3)	Communication experienced between ENs and GPs, ENs and EN Team leader, within the EN team as well as between ENs and patients/relatives To what extent ENs feel confident in communicating with GPs regarding -symptom management -need for delegated services To what extent ENs feel confident in communicating with patients/relatives regarding -symptom management -coping with daily life -emotions (e.g. anger, grief)	✓	✓
		Principal Investigator	1	Interviews (2)	Experienced internal and external communication Barriers and facilitators		✓
		GPs	All GPs	Interviews (2), questionnaires (3), focus groups (2)	Communication experienced between ENs and GPs To what extent GPs experience ENs in communication in terms of -professional competence -patient-centeredness -interprofessional communication -solution-oriented approaches	✓	✓
		Patients	2–4	Interviews (6)	Experienced communication (incl. difficulties, contents of communication)		✓
		Relatives	2–4	Interviews (6)	Experienced communication (incl. difficulties, contents of communication)		✓
		Other health care professionals from other health care services involved in HiH patient care	5	Interviews (2)	Experienced communication, ways of communication		✓
	Trust	ENs	10	Interviews (3), focus groups (3), questionnaires (3)	Indication for a trusting interprofessional relationship between ENs and GPs	✓	✓
		GPs	All GPs	Interviews (2), focus groups (2), questionnaires (3)	Indication for a trusting interprofessional relationship between GPs and ENs	✓	✓
		Patients	2–4	Interviews (6)	Indications for a trusting interpersonal relationship between ENs and patients		✓
		Relatives	2–4	Interviews (6)	Indications for a trusting interpersonal relationship between ENs and patients/ ENs and relatives		✓

*EN* Expert nurse, *ENC* Expert nursing centre, *EN Team leader* Team leader of the e xpert nurses, *ePA* Electronic patient documentation system, *GP* General practitioner, *HiH* HandinHand study, *Quant.* Quantitave, *Qual.* Qualitative
4. Context
  In this section, several external factors that may influence the intervention will be explored, including aspects on the macro, meso, and micro levels. As recruitment for the HiH study started during the emergence of the worldwide COVID19 pandemic, this as a relevant context factor in the process evaluation will be included. Details of data collections pertaining to the process domain of “context” are described in Table 5.
  - Macro level
    Important aspects to consider here will be the German health system including legal frameworks pertaining to the professional practice of health care professionals. An attempt will be made to reflect on the interprofessional culture in healthcare in Germany. Also, structural features and geographic conditions that impact on the intervention will be considered. This will include the innovative feature of the ENC as well as the rural nature of the region.
  - Meso level
    At the meso level, the following institutions and aspects will be of interest in describing the context:
    Equipment, staffing and structure of the ENC
    Characteristics of participating medical practices and current main challenges for GP practices in general
    Unique features of setting, in which the intervention will be implemented (primary care setting)
    Number of hospital admissions, rehospitalisation and potentially avoidable hospitalisations in the catchment area in relation to the patient group targeted by the intervention
    Description of type and availability of services in the catchment area (community-based support services and allied health services)
    Contents of the EN’s university course where the ENs complete their accompanying studies as well as investigation of the perspective of university representatives concerning further development of the professional role of nurses
    Examination of the perspective of representatives of health insurances, considered to be of great importance in the implementation of new health care structures.
  - Micro level
    To develop a good understanding of the micro-level context, the characteristics and other relevant aspects of potential participants (patients, relatives, nurses with expanded roles, GPs) on group level will be described. Since medical assistants with further training also work in primary care, their role and qualifications in comparison to ENs, as well as the possible overlaps in this area will also be described. In addition, other health care professionals involved in the project will be asked about factors they see as facilitating and hindering the implementation of the new role and the ENC.
  - COVID19
    The COVID-19 pandemic and related measures unexpectedly have become a relevant context factor for this study. Therefore, the question will be addressed to what extent the pandemic influences the medical care of patients, the work of the ENs and the ENC as well as the collaboration with other health care professionals and the project as a whole. COVID-19 has been identified as a contextual factor after completion of the logic model and is therefore not included in the model.
5. Process outcomes
  The central topic of nursing role and competency development will be considered in the domain of process outcomes. Longitudinal data on the experience of participants over time will also be collected. Details of data collections pertaining to the domain of process outcomes are described in Table 6.
  - Nursing role and competency development over time
    To explore role and competency development, the perspective of ENs as well as EN team leaders and PTHV representatives who are each involved in supporting role and competency development will be investigated. The perspective of the GPs and other health care professionals will be of interest with regard to changes in the delegation of tasks. Patients and relatives will be asked to what extent they can describe a change in the perception of the EN's role. Furthermore, it will be investigated to what extent different participants of the study (ENs, EN team leader, representative of the PTHV, GPs, and the principal investigator) and stakeholders (representatives of a health insurance) describe barriers and facilitating factors regarding role and competence development.
  - Experiences of participants over time
    Of particular interest here will be the perspective of patients and relatives regarding the perceived care by ENs, differences and similarities compared to care provided by GPs and other health care professionals, and to what extent they perceive changes in their health status as well as the perceived empathy by ENs [24]. Furthermore, core participants as well as the principal investigator will be asked about perceived benefits and disadvantages for patients and relatives, GPs and ENs, for others, the region and the health care system as a whole [10].
    In addition, all participants will regularly be asked about their recommendations for long-term implementation and what they see as hindering and facilitating factors.

**Table 5:** Data collection in the process domain “context”

Process domain indicator	Topics	Data sources	N (planned number of persons per data collection)	Data collection methods (number of planned data collections where applicable)	Operationalisation/ Parameters	Data type	
						Quant	Qual
a. Macro level	German health care system	Ministry of Health, Federal and State Offices of Statistics	n.a	File review	Basic structure of the German health care system		descriptive
	Legal framework	Legislation relating to primary care and the practice of the nursing profession	n.a	File review	Summary of key features and legislation surrounding nursing practice and health care - Nursing care - Primary care - Delegation - Ambulatory care		descriptive
	Geographical conditions	Federal Statistical Office and Statistical Office of Rhineland-Palatinate	n.a	File review	Geographical conditions and unique features of the catchment area		descriptive
	Structural context	Federal Statistical Office and Statistical Office of Rhineland-Palatinate Ministry of Social Affairs, Labour, Health and Demography of Rhineland-Palatinate Association of Statutory Health Insurance Physicians Nursing Council	n.a	File review	Health care and health educational structures in Rhineland-Palatinate - Characteristics of hospitals, nursing homes, medical practices and ambulatory care services - Number of nursing/medical degree programmes; number of vocational nursing schools - Number of practicing nurses and GP		descriptive
		Relevant literature	n.a	Literature review	Potential fit of new care models into existing structures incl. best-practice examples, barriers and facilitators		descriptive
		Principal investigator	1	Interviews (2)	Potential fit of the HandinHand care model into existing health care structures, barriers and facilitators		✓
		EN team leader	2	Interviews (3)			✓
		ENs	10	Interviews (3)			✓
		GPs	All GPs	Interviews (2)			✓
		PTHV	2	Interviews (2)			✓
		Health insurance	2	Interviews (2)			✓
		Other health care professionals from other health care services involved in HiH patient care	5	Interviews (2)			✓
		EN team leader	1	Questionnaires (3)	Characteristics of the catchment area		✓
	Interprofessional culture	Relevant literature	n.a	Literature review	Summary of the main features that influence cooperation between GPs and nurses in general		descriptive
		Narratives	n.a	E-Mail correspondence, annual reports, case studies			descriptive
		Other health care professionals from other health care services involved in HiH patient care	5	Interviews (2)	Perception of interprofessional cooperation in primary care		✓
b. Meso level	EN Centre	EN team leader	1 2	Questionnaires (3), interviews (3)	Description of equipment, staffing and structure of the ENC	✓	✓
	Setting	EN	10	Interviews (3)	Description of features unique to settings (patients home rather than nursing homes)		✓
		Narratives	n.a	E-Mail correspondence, annual reports, case studies	Description of features unique to settings/region (patients home rather than hospitals)		descriptive
		Relevant literature	n.a	Literature review	Description of features unique to settings/region (patients home rather than hospitals)		descriptive
	GP/specialist practice	GPs	All GPs	Questionnaires (3)	Characteristics of participating medical practices - number of patients at each practice - staffing and skill mix	✓	
		Association of Statutory Health Insurance Physicians Federal and State Offices of Statistics	n.a	File review	Current main challenges for GP practices - Shortage of GPs - Demographic developments - Ageing society with increasingly complex care needs		descriptive
		Relevant literature	n.a	Literature review	Strategies for dealing with the challenges of an ageing society, implications for GPs and care provision		descriptive
	Hospital	Federal Statistical Office and Statistical Office of Rhineland-Palatinate; Ministry of Social Affairs, Labour, Health and Demography of Rhineland-Palatinate; Other relevant data sources	n.a	File review	Number of hospitalisations, re-hospitalisations and potentially avoidable hospitalisations with regard to people with chronical diseases Discharge management		descriptive
	Community-based support services	EN team leader	1	Questionnaires (1)	Type and availability of community-based support services (e.g. hospice care, meals on wheels, support groups)	✓	
	Allied health services	EN team leader	1	Questionnaires (1)	Type and availability of allied health services	✓	
	PTHV	Course manual	n.a	Document review	Degree programme overview - degree structure - content of papers		descriptive
		Paper coordinators	2	Interviews (2)	Perspective of the PTHV with regard to - role development of nurses in general and in context of the project in particular the establishment of the ENC in particular and the HiH intervention in general Extend to which degree personnel supports role development (e.g. clinical reflection, supervision, exemplar writing)		✓
	Health insurances	Health insurance representatives	2	Interviews (2)	Motivation for participation and perspectives on innovative care models with regard to primary care structures in general and the project intervention particular		✓
		Federal Joint Committee/ Narratives	n.a	Database search/E-mail correspondence with professional networks	Overview of other innovative projects addressing health care restructure in primary care funded by the Ministry of Health		descriptive
c. Micro level	Patients	Relevant literature	n.a	Literature review	Description of - expectations for health care in general - attitudes and expectations of health care by GPs compared to health care provided by nurses		descriptive
	Relatives	Relevant literature	n.a	Literature review	Description of - patient/caregiver dyad - needs for support services for informal caregivers - attitudes and expectations of health care by GPs compared to health care provided by nurses		descriptive
	ENs	ENs	10	Interviews (1), questionnaires (1)	Characteristics of EN -professional experience Description of attitudes and expectations of the project/position	✓	✓
		Job description	n.a	Document review	Description of formal requirements		descriptive
	EN team leader	EN team leader	2	Interviews (2)	Attitudes and expectations of the project		✓
		Relevant literature	n.a	Literature review	Challenges for team leaders regarding change management in health care Challenges of implementing a new role		descriptive
		Narratives	n.a	E-mail correspondence with e.g. the EN Team leader/case studies			✓
	GPs	Relevant literature	n.a	Literature review	Summary of main challenges of GPs in Germany		descriptive
	Medical assistants (NäPa’s)	Federal Medical Council/ National and Federal Association of Statutory Health Insurance Physicians	n.a	File review	Role description and qualifications Overlap with ENs		descriptive
	Other health care professionals	Other health care professionals from other health care services involved in HiH patient care	5	Interviews (2)	Views on barriers and facilitators for the establishment of the new role		✓
COVID-19	Influence of the COVID-19 pandemic	ENs	10	Interviews (3)	Influence on the EN role and the ENC		✓
		EN team leader	2	Interviews (3)	Influence on the EN work and the EN management		✓
		Principal investigator	1	Interviews (2)	Influence on the project and the EN work		✓
		GPs	All GPs	Interviews (2)	Influence on the project and the EN work		✓
		Patients	2–4	Interviews (6)	Influence on own health and care situation		✓
		Relatives	2–4	Interviews (6)	Influence on the patients’ health and care situation		✓
		PTHV	2	Interviews (2)	Influence on the EN studies and their work		✓
		Health Insurances	2	Interviews (2)	Impact on the project		✓
		Other health care professionals from other health care services involved in HiH patient care	5	Interviews (2)	Impact on collaboration		✓

*EN* Expert nurse, *ENC* Expert nursing centre, *EN Team leader* Team leader of the e xpert nurses, *ePA* Electronic patient documentation system, *GP* General practitioner, *HiH* HandinHand study, *Quant.* Quantitave, *Qual.* Qualitative

**Table 6:** Data collection in the domain “process outcomes”

Process domain indicator	Topics	Data sources	N (planned number of persons per data collection)	Data collection methods (number of planned data collections where applicable)	Operationalisation/Parameters	Data type	
						Quant	Qual
Role and competence development of the ENs	Development over time	ENs	10	Interviews (3), questionnaires (4), focus groups (2)	Description of whether and how the role and competence development has taken place Retrospective perception of the role and role development Predominant issues of the ENs at different times Presumption of the ENs how the role will develop		
		EN team leader	2 1	Interviews (3), questionnaires (3)	Changes in the perception of the ENs competencies and role clarity	✓	✓
		GPs	All GPs	Interviews (2), questionnaires (3), focus groups (1)	Changes in the perception of the ENs competencies Changes in delegation of tasks	✓	✓
		PTHV	2	Interviews (2)	Changes in the perception of the ENs competencies and role clarity		✓
		Other health care professionals from other health care services involved in HiH patient care	2	Interviews (2)	Changes in the perception of the ENs competencies Perceived changes in delegation of tasks		✓
		Patients	2–4	Interviews (6)	Changes in the perception of the EN role		✓
		Relatives	2–4	Interviews (6)	Changes in the perception of the EN role		✓
	Barriers and facilitators	ENs	10	Interviews (2), focus groups (1)	Perceived barriers and facilitators the development of role and competencies		✓
		EN team leader	2	Interviews (3)	Barriers and facilitators in the support of role and competence development		✓
		Health insurance	2	Interviews (2)	Barriers and facilitators regarding the development of new nursing roles in general		✓
		PTHV	2	Interviews (2)	Barriers and facilitators in teaching necessary competencies Barriers and facilitators in the support of role and competence development		✓
		GPs	All GPs	Interviews (2)	Barriers and facilitators in the support of role and competence development		✓
		Principal Investigator	1	Interviews (2)	Challenges of role and competence development		✓
Experiences of the participants							
**Patients**	Perception of care delivered	Patients	2–4	Interviews (6)	Overall perception, aspects of trust, shared decision-making, empowerment Differences to and similarities with other health professionals Perceived changes in health status		✓
	Benefits and disadvantages	Patients	2–4	Interviews (6)	Perceived benefits and disadvantages of ENs care		✓
	Empathy	Patients	All patients	Process Outcome Questionnaires (RWI)	Perceived empathy (empowerment, shared decision-making)	✓	
**Relatives**	Perception of care delivered	Relatives	2–4	Interviews (6)	Overall perception, aspects of trust, shared decision-making, empowerment Differences to and similarities with other health professionals Perceived changes in health status		✓
	Benefits and disadvantages	Relatives	2–4	Interviews (6)	Perceived benefits and disadvantages for themselves		✓
**GPs**	Perception of care delivered	GPs	All GPs	Interviews (2), focus groups (1), questionnaires (3)	Overall perception, unique contribution of ENs, shared decision-making Differences to and similarities with other health professionals	✓	✓
	Benefits and disadvantages	GPs	All GPs	Interviews (1), focus groups (1)	Perceived benefits and disadvantages for themselves, patients & relatives, others, the region, the health care system as a whole		✓
Principal investigator	Benefits and disadvantages	Principal investigator	1	Interviews (2)	Perceived benefits and disadvantages for patients & relatives, others, the nursing profession, the region, the health care system as a whole		✓
ENs	Benefits and disadvantages	ENs	10	Interviews (1), focus groups (1)	Perceived benefits and disadvantages for patients & relatives, others, the nursing profession, the region, the health care system as a whole		✓

*EN* Expert nurse, *ENC* Expert nursing centre, *EN* Team leader Team leader of the expert nurses, *ePA* Electronic patient documentation system, *GP* General practitioner, *HiH* HandinHand study, *Quant.* Quantitave, *Qual.* Qualitative

### Data analysis

Quantitative data will be analysed descriptively using IBM SPSS Statistics (IBM Corp. Released 2020, IBM SPSS Statistics for Windows, Version 27.0.0.0). Qualitative data will be inductively-deductively analysed using QCAmap [22] and MAXQDA Standard 2020 (Release 20.0.8, VERBI GmbH Berlin). A qualitative thematic framework analysis will be conducted [25]. All data will be analysed iteratively so that emerging themes from early interviews, focus groups or questionnaires can be explored in later ones. As recommend in the MRC Framework, qualitative data will be combined with quantitative data relating to key process variables and process outcomes where it is to be expected that they influence the effect and function of the intervention [23]. The logic model will serve as framework for assigning the different perspectives and findings to the constructs of implementation, the mechanisms of impact, the context and the outcome parameters. Narratives will be integrated after an initial analysis of qualitative data has been completed.

## Discussion

The comprehensive process evaluation will enable to obtain important indications at different levels as to which hindering and facilitating factors need to be taken into account in the future. Because of the complexity and the need to gain the relevant insights, the challenge will be to limit the analyses to the core questions and handle the amount and potential incommensurability of data. To overcome this challenge, the logic model will provide orientation and structure and will help to focus [23].

The MRC framework addresses further key recommendations for process evaluations [23] that need to be discussed. As there will be a separation between the process and outcome evaluation, the methods and timing of the data collections with the institutions responsible for the outcome evaluation (RWI) were discussed in advance and synergies were identified. The cooperation and communication is constructive and trusting. In addition, a representative of the RWI is a permanent guest at the meetings of the advisory board. Advisory support was provided by the research team in the development of the intervention. If required, different aspects of the intervention and nursing sciences in general are discussed between the EN team leader and the research team. As the research team is aware of the need to remain credible evaluators, they will reflect their role and the potential impact on the research process regularly within the research team.

As the intervention, for which the TIDieR instrument was used [12], was discussed in detail in the project advisory board and between the HiH core team and the research team, a precise description of the intervention exists. Changes made after four months were recorded accordingly.

The recommended entry level for ANP is Master-level tertiary education (ICN, [13]). Given the late development of Master-level tertiary education for nurses and that there are still hardly any APNs working regularly in primary care in Germany, the implementation of the ENC comprising ENs with an additional tertiary nursing education at Bachelor-level can be an important intermediate step to advance the development of nurses with expanded roles. At this level, nurses are provided with the skills and competencies required for evidence-based expanded practice. At the same time, the roles for this type of practice will need to be developed and evaluated in terms of scope, effectiveness, and integration into the existing healthcare system. Such roles could pave the way for an expanded, autonomous nursing practice towards ANP implementation in Germany to improve care for older people with chronic illnesses. As this complementary role of nurses does not yet exist in this setting, there will be a special focus on the development of the role of the ENs and their competencies as well as their scope of practice within the process evaluation.

The chosen methodological approach has several advantages. The applied mixed methods approach allows to measure key variables quantitatively as well as qualitative. This will enable to build upon quantitative findings more precisely in interviews or focus groups. In addition, all core participants and relevant stakeholders will be interviewed several times during the course of the project, so that the perspectives at different levels can be considered. To avoid collecting unnecessary data, smaller purposively selected samples have been chosen.

It will be essential to integrate relevant unpredictable contextual factors such as the COVID-19 pandemic into the data collection, which is why all interviewees will be asked about the influence of the pandemic. In the course of data collection, some planned data collections needed and will need to be postponed due to the COVID-19 pandemic and some data collection methods were and will be adapted, i.e. changing from face-to-face to video interviews or from focus groups to individual interviews.

## Data Availability

Due to the conditions of informed consent with participants, the datasets generated and analysed during the process evaluation will not be publicly available. Please contact the corresponding author in case of reasonable requests.

## Abbreviations

ANP: Advanced Nursing Practice
APN: Advanced Practice Nurse
ePA: Electronic Patient Documentation System
EN: Expert Nurse
ENC: Expert Nursing Centre
EN Team leader: Team leader of the expert nurses
GP: General Practitioner
HCP: Health Care Professionals
HiH: HandinHand
n.a.: Not applicable
PI: Principal Investigator
PTHV: Philosophisch-Theologische Hochschule Vallendar (Catholic University), Faculty of Nursing Science, Germany
Qual.: Qualitative
Quant.: Quantitative
RWI: RWI—Leibniz Institute for Economic Research
TIDieR Checklist: Template for Intervention Description and Replication
